# Long-term exposure of CdTe quantum dots on PC12 cellular activity and the determination of optimum non-toxic concentrations for biological use

**DOI:** 10.1186/1477-3155-8-7

**Published:** 2010-03-25

**Authors:** Babu R Prasad, Natalia Nikolskaya, David Connolly, Terry J Smith, Stephen J Byrne, Valérie A Gérard, Yurii K Gun'ko, Yury Rochev

**Affiliations:** 1 National Centre for Biomedical Engineering Science, National University of Ireland, Galway, Ireland; 2 CRANN and The School of Chemistry, Trinity College Dublin, Dublin 2, Ireland

## Abstract

**Background:**

The unique and tuneable photonic properties of Quantum Dots (QDs) have made them potentially useful tools for imaging biological entities. However, QDs though attractive diagnostic and therapeutic tools, have a major disadvantage due to their inherent cytotoxic nature. The cellular interaction, uptake and resultant toxic influence of CdTe QDs (gelatinised and non-gelatinised Thioglycolic acid (TGA) capped) have been investigated with pheochromocytoma 12 (PC12) cells. In conjunction to their analysis by confocal microscopy, the QD - cell interplay was explored as the QD concentrations were varied over extended (up to 72 hours) co-incubation times. Coupled to this investigation, cell viability, DNA quantification and cell proliferation assays were also performed to compare and contrast the various factors leading to cell stress and ultimately death.

**Results:**

Thioglycolic acid (TGA) stabilised CdTe QDs (gel and non - gel) were co-incubated with PC12 cells and investigated as to how their presence influenced cell behaviour and function. Cell morphology was analysed as the QD concentrations were varied over co-incubations up to 72 hours. The QDs were found to be excellent fluorophores, illuminating the cytoplasm of the cells and no deleterious effects were witnessed at concentrations of ~10^-9 ^M. Three assays were utilised to probe how individual cell functions (viability, DNA quantification and proliferation) were affected by the presence of the QDs at various concentrations and incubation times. Cell response was found to not only be concentration dependant but also influenced by the surface environment of the QDs. Gelatine capping on the surface acts as a barrier towards the leaking of toxic atoms, thus reducing the negative impact of the QDs.

**Conclusion:**

This study has shown that under the correct conditions, QDs can be routinely used for the imaging of PC12 cells with minimal adverse effects. We have found that PC12 cells are highly susceptible to an increased concentration range of the QDs, while the gelatine coating acts as a barrier towards enhanced toxicity at higher QD concentrations.

## Background

Semiconductor nanoparticles or Quantum Dots (QDs) have been widely touted as new replacements for traditional dyes for the imaging of living cells and tissues. Due to their extremely small size QDs can, *via *specific and non-specific pathways penetrate and label both the exterior and interior of numerous cell types [1-7]. They are highly resistant to photobleaching [2,8-10] and their broad absorption ranges allow for their excitation and multiplexed detection across a wide spectrum of wavelengths [11-14].

Minute changes in the radius of QDs manifests as visible colour changes of the QDs in solution. This property may lead to their potential use as simultaneous multiple colour labels [15-17] The difference in size can also affect their uptake may lead to alterations in cellular activity and cytotoxicity [18,19].

Our studies are focussed on the analysis of PC12 cells which have the ability to be differentiated into neurons upon treatment with nerve growth factors (NGF). The application of QDs to neuroscience specific fields is currently emerging [20-25] and various groups have investigated the specific labelling of neurons with QDs. Nerve growth factors were QD tagged by Vu *et al *[26], QD micelles were up taken by rat hippocampal neurons as shown by Fan *et al *[27], while various antibody and peptide labelled QDs have also been explored [6,20,28-32]. However, advances in molecular medicine require the safe detection of individual biomolecules, cell components and other biological entities. One significant problem with QDs is their heavy metal composition [33-35], which has given genuine cause for concern due to their potential cytotoxicity [33,35,36]. In an effort to combat this problem, much research has been conducted into the mechanisms that result in QDs acting as toxic agents once exposed to a cellular environment [37-43] and ways of reducing their toxicological impact *via *non-toxic coatings [44].

While QDs have been investigated with a large variety of cell lines and types; more recently, in search of new neurotherapeutic and neuroprosthetic strategies, QDs have been explored to manipulate and create active cellular interfaces with nerve cells [19,20]. However, the application of such entities to neuron cell imaging is limited and while QDs have been used for cell labelling experiments, little work has been undertaken into measuring the ranges of neuron cell response over long time scales upon their perturbation by the QDs.

The purpose of the study was to explore the potential for labelling of undifferentiated Pheochromocytoma 12 (PC12) cells with gelatinised and non-gelatinised TGA capped CdTe QDs. We have studied serial co-incubations of 24, 48 and 72 hours and analysed the effect of three factors namely concentration, co-incubation time and surface modification in parallel to three assays measuring cell viability, proliferation and DNA quantification. Although shorter incubation periods have been used by some groups to investigate the toxicity [42,45], long term exposure is more reliable. There are a number of studies which have investigated the toxicity of QDs for 24 hour co-incubations and demonstrated that increasing concentrations increase cell toxicity significantly [23,45-48].

## Results and Discussion

### Optical characteristics

**Figure 1:**
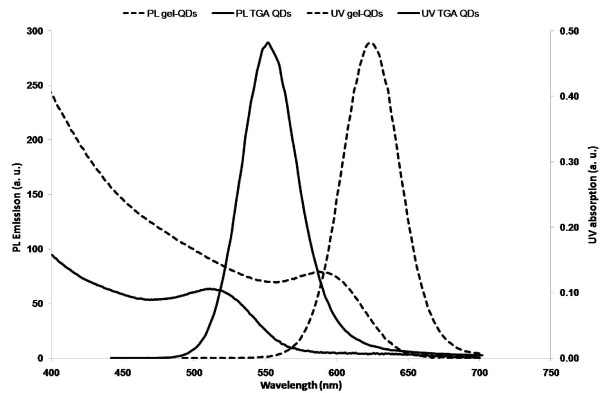
**Absorption and emission spectra**. UV-vis absorption and fluorescence emission spectra (λ_em _450 nm) of the differently sized (~2.5 nm - solid line & ~4.5 nm - dashed line) QDs synthesised and co-incubated with the PC12 cells.

The two types of QDs utilised (gel and non-gel) were synthesised using a modification of a previously published procedure [49]. This synthetic route allows for the production of highly luminescent and crystalline CdTe QDs. Briefly, H_2_Te gas was bubbled through an basic aqueous solution containing Cd(ClO_4_)_2_6H_2_O, thioglycolic acid (TGA) stabiliser and dissolved gelatine where appropriate. The resultant non-luminescent mixture was heated under reflux. The crude solutions were purified *via *size selective precipitation and individual fractions were characterised by UV-vis absorption and photoluminescence (PL) emission spectroscopy (λ_ex _425 nm). Prior to initiating cell culturing experiments, the QDs were further purified using sephadex (G25). This enabled us to remove any residual un-reacted moieties that may have been present from the original crude solution. Two differently sized batches of QDs (for both gel and non-gel QDs) were synthesised to allow us to investigate if the additional parameter of QD size had any impact on cell response. Figure 1 shows the typical absorption and emission profiles indicative of aqueous CdTe QDs. As there are no differences in the spectral characteristics of gel and non-gel QDs, one spectrum indicative of each size is shown for clarity.

The spectra shown in Figure 1 highlight the well resolved emission and absorption characteristics of the QDs. Narrow emission spectra (<40 nm full with half maximum [FWHM]) indicate <5% particle size distributions throughout. Gelatine was introduced during the synthesis of the QDs and its presence while altering QD growth rates and QYs [44], does not significantly alter the size distribution of the QDs and acts primarily as a co-capping agent.

Quantum yields (QYs) for the solutions (measured against Rhodamine 6G) were ~25% for the non-gel and ~35% for the gel QDs. As the presence of uncapped surface atoms provides alternate pathways for the non-radiative recombination of photons, the difference in QYs indicate the highly effective capping qualities of the gelatine.

**Figure 2:**
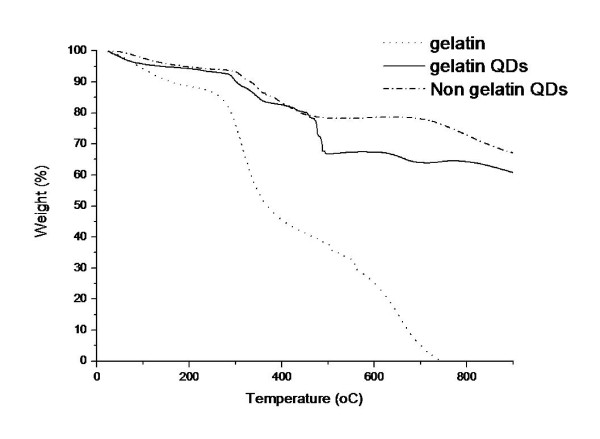
**Thermogravimetric analysis**. Graph showing the percentage weight loss for the QD and gelatine samples upon heating to 900°C.

To examine the quantity of gelatine on the QD surface we analysed the QDs using thermogravimetric analysis (TGA). This process involves burning the sample to be examined and measuring the weight loss against temperature (Figure 2).

For TGA experiments, each sample was first dried and subsequently weighed. The sample was then heated (from 30 to 900°C at a rate of 10°C/min) and as each component was burned off, the weight changes were recorded. For both types of QDs several steps can be seen. The initial drop in weight is due to the removal of water molecules. Following on, we can now see the weight loss due to the removal of the organic molecules from the QD surface. We can see a clear difference in the profiles of the two QD types. The gel QDs show an additional weight loss (~10%) at ~500°C compared to the non-gel QDs thus indicating the presence of excess organic groups that we are attributing the gelatine coating. We have also analysed the behaviour of gelatine under the same conditions as an additional guide.

**Figure 3:**
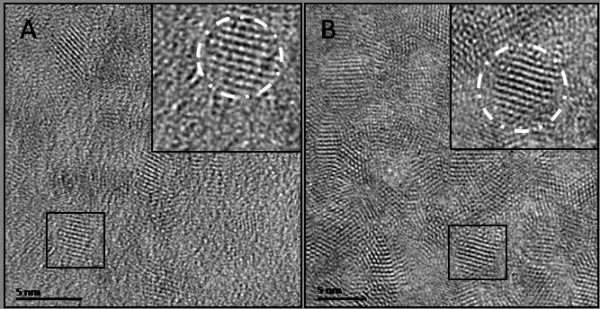
**HRTEM QD characterisation**. HRTEM images of (A) non-gel (~2.5 nm) and (B) gel (~4.5 nm) capped CdTe QDs. (Inserts are blown up images of highlight QDs).

High resolution transmission electron microscope (HRTEM) images were taken to examine the structure and morphology of the two differently sized types of QDs (Figure 3).

HRTEM images of the different sized QDs show the highly crystalline nature of both the gel and non-gel QDs (Figure 3). Lattice spacings are in agreement with those expected for the (111) plane of cubic zinc blend CdTe [50]. We have previously shown that although the presence of gelatine during the synthesis of the QDs can influence the rate of QD growth and QY [44], it does not seem to alter the physical structure of the QDs. Consequently, as can be seen from the resulting QY's, the gelatine must act solely as a co-capping agent for the protection of the QD surface and the reduction of non-radiative transitions. The incorporation of gelatine during the QD synthesis results in smaller QDs being produced under the same conditions compared to non-gel QDs but does not seem to alter or influence the size distribution with the particle ensemble. Following size selective purification, size distributions for spectroscopically similar gel and non gel samples were comparable with the only noticeable difference being their respective QYs.

The influence of this additional exterior coating upon uptake and any induced toxicity were some of the properties we wished to explore with the PC12 cells.

**Figure 4:**
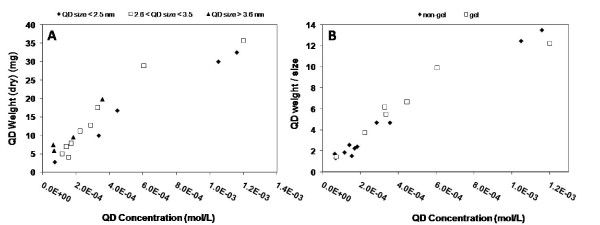
**QD weight versus concentration profile**. Graphs illustrating the relationship between measured QD concentration and QD powdered weight (A) and QD powdered weight/size (B).

We have also conducted a number of experiments in an effort to empirically relate the actual mass (mg of QDs per ml) of the QDs used in solution to their determined concentration [17]. (note: QDs treated as individual molecules for the purpose of concentration determination). Several different batches of gel and non-gel QDs were dried under rotary evaporation. A measured amount of the resulting QD powder was then weighed and dissolved in exactly 1 ml of purified water. The molar concentration was then determined for each individual batch [17]. Figure 4 illustrates the relationship between QD weight and molar concentration (M) for our QDs used.

As expected there is a linear relationship between measured QD concentration and powdered weight. This allows us to postulate as to the concentration (mg/ml) of QDs that we have used throughout our experimental analysis. We have also included a plot of concentration against weight/size, to give a fuller empirical relationship for the system under investigation. It must be noted that as the QDs are dried from solution (although fully purified), there is the possibility that QD degradation may occur which increases the experimental error with regards to concentration, but overall it does give us a good general indication.

**Figure 5:**
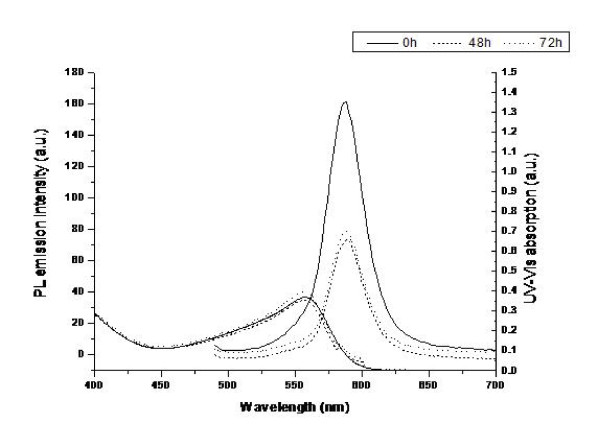
**QD interactions with cell culture medium**. Evolution of UV-vis absorption and PL emission spectra (λ_exc _480 nm) of non-gel QDs in cell culture medium over time.

**Figure 6:**
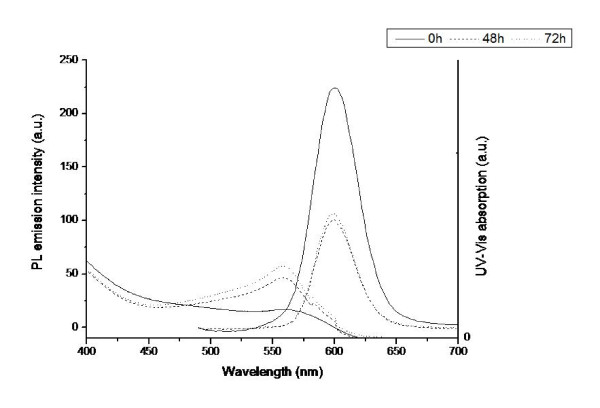
**QD interactions with cell culture medium**. Evolution of UV-vis absorption and PL emission spectra (λ_exc _480 nm) of gel QDs in cell culture medium over time.

To investigate any possible degradation of the QDs without the presence of the PC12 cells, we carried out a number of experiments to analyse the effect of co-incubating the QDs with only the cell culture medium (Figure 5 and 6).

Figures 5 and 6 show the evolution of the UV-vis absorption and PL emission (λ_ex _480 nm) spectra of non-gel and gel QDs respectively in cell culture medium over time. The unusual shape of the UV spectra is due to the interference caused by the culture medium. This was used as a background throughout but its effect could not be completely removed. For the gel QDs at 0 hours, the UV spectrum is as expected but as the incubation times increased, the effect of the medium became apparent. Most importantly however, the UV spectra of both QD types remain consistent and do not drop even after 72 hours. This indicates that the core structures of the QDs remain intact and that no significant degradation to the QDs themselves is occurring. If degradation were occurring, the baseline would rise as the QD begin to precipitate from solution and the absorbance and structure of the spectrum would decrease significantly. This core stability is further corroborated by the PL spectra which show an initial drop after 48 hours, but stability thereafter. This quenching of the emission properties of the QDs is common when recorded in the presence of biological media.

Previously, we have investigated the effect of QD and protein charge on QD spectra and cellular interactive characteristics [51]. As the medium contains serum, these spectral changes can be attributed to the interaction of the various proteins present with the QD surface. These interactions do not lead to the degradation of the QDs, but do provide alternate pathways for radiative recombination, thus resulting in lower fluorescence intensities. If the QDs begin to degrade following cellular uptake, resulting in leeching of the core atoms; it must be attributable to the harsh intracellular conditions that the QDs face within the cytoplasm.

Our next aim was to analyse the effect of the QDs on cell behaviour and morphology also to then investigate any alterations to cell proliferation, viability and DNA quantification using pre-determined assays over extended co-incubation times.

#### 1. Uptake of QDs and their effect on cell morphology

**Figure 7:**
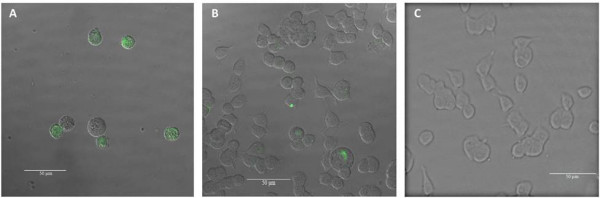
**Confocal image**. Fluorescent confocal image and corresponding differential interference contrast (DIC) images of PC12 cells exposed to a 10^-7^M concentration of QDs (A), 10^-9 ^M concentration of QDs (B) and a control sample with no QDs (C) following 72 hours of co-incubation. Scale bar = 50 μm.

**Figure 8:**
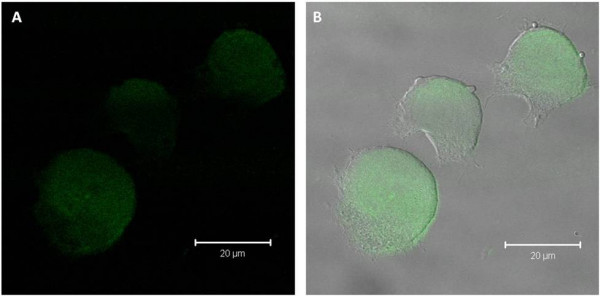
**Confocal Image**. Fluorescent confocal image of PC12 cells exposed to a 10^-9 ^M concentration of QDs (A) and corresponding differential interference contrast (DIC) image (B) with A overlaid following 72 hours of co-incubation [scale bar = 20 μm].

**Figure 9:**
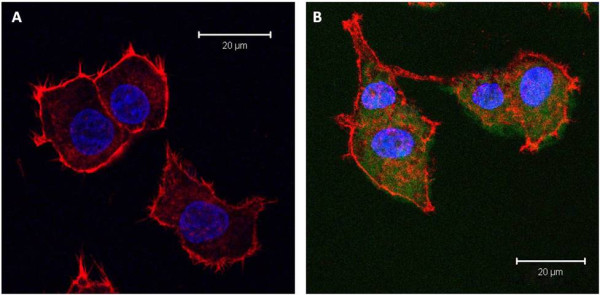
**Confocal images**. Fluorescent confocal images to illustrate the morphology of the actin stained PC12 cells with no QDs (A) as a control and PC12 cells exposed to the QDs (B) [conc. 10^-9 ^M] following 72 hours of co-incubation. [Scale bar = 20 μm].

Stock gel and non-gel QD solutions (10^-4 ^M) [17] were diluted to a range of concentrations (10^(-7)-(-9) ^M) and incubated with the cells as described in the experimental section. Confocal images were taken to visually inspect QD uptake, localisation and cell morphology following incubation (Figures 7, 8, 9).

Figure 7, panels A and B show PC12 cells following 72 hours of co-incubation with 10^-7 ^M and 10^-9 ^M concentrations of QDs respectively. In panel A, the cells were seen to be rounded and floating in the nutrient rich medium. This contrasts the morphology of the cells in panel B and the control cells (panel C), which were attached to the culture plate and polygonal in shape. It can be noted that as QD concentrations were reduced, the effect on the cell morphology was eliminated and the cells were morphologically identical to the control cells (Figure 7, panels B and C). Although some earlier studies [23,48] have shown similar concentration dependence, there is no study investigating the effect on cell morphology at the extended time periods of 48 and 72 hours [45]. Green fluorescence in the PC12 cells is due to QDs localisation in the cytoplasm.

Figure 8 shows the fluorescent image (panel A) and overlaid corresponding differential interference contrast (DIC) image (panel B) of the PC12 cells treated with a 10^-9 ^M concentration of QDs following 72 hours of co-incubation. The QDs are found to be located within the cytoplasm of PC12 cells.

To enhance visualization, the nucleus and cellular membrane have been actin stained with blue and red colour respectively (Figure 9). The QDs (green luminescence) are visualized predominantly in the cytoplasm and their presence even after a 72 hour co-incubation in this region, does not seem to significantly perturb the cells. The cell morphology does not change when evaluated against the controls.

These initial observations illustrate the effect of changing QD concentration on cell survival and morphology and to further investigate cell behaviour, several assays were used to study the effect on cell proliferation, growth and metabolic activity.

#### 2. Effect of QDs on cellular activity

The consequence of co-incubating classical molecules on the cell viability can be reliably predicted using single assays [52], however, the dynamics of nanomaterials are not as comprehensively understood and hence drawing conclusions from single cell viability assays can be misleading. As such additional assays are required to give a more comprehensive analysis when determining nanoparticle toxicity for risk assessment [52].

Consequently, alamarBlue (metabolic activity), PicoGreen (total DNA quantification) and ELISA BrdU (colorimetric assay for quantification of proliferating DNA) assays were run to analyse the effect of different QD concentrations, type and size following 24, 48 and 72 hour co-incubations with the PC12 cells.

The red/orange labels serve to differentiate the various QDs by size [~2.5 nm (orange) and ~4.5 nm (red)] and were used to investigate if the measured cell responses were in any way size dependant. The gel/non-gel label refers to the presence of gelatine during the synthesis of the QD and these different QDs were analysed to investigate the influence that gelatine imparts on the QD induced cell toxicity.

The changes in luminescence intensity measured in response to the introduction of QDs to the cell cultures throughout all of our experiments can be solely attributed to direct interactions of the staining dyes upon entering the cells. Energy transfer to the dyes can be ruled out *via *a number of routes. Firstly, the dyes and QDs enter different regions of the cells and as such cannot interact directly on the scale required for FRET or other energy transfer phenomena. Secondly, the intensity (arbitrary units) of the dye emission is of the order of ~10^3 ^while the QDs display ~10^2^. Thus, any energy transferred to the dye would be of an order of magnitude lower and would have a minimal effect on the emission intensity. Negative and background controls in our experiments also substantiate this fact.

#### 2.1 AlamarBlue Assay

**Figure 10:**
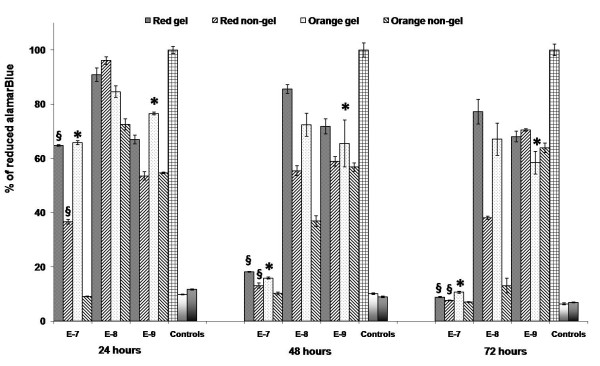
**AlamarBlue histograms**. AlamarBlue assay at 24, 48 and 72 hours showing the viability of PC12 cells after treatment with varying concentrations [10[[(-7)-(-9)] M] of the gel and non-gel QDs. From left to right, controls [positive, negative, background] are also shown. §denotes examples of statistical significance due to effect of gelatine, * denotes examples of statistical significance due to effect of concentration using a one- way ANOVA (p < 0.05) by Tukey's mean comparison.

Viability of the PC12 cells, for different concentrations, sizes and types of QDs was investigated with an alamarBlue assay and the results graphed in Figure 10. This is a non-destructive assay and allows for the cells to be further utilised following analysis.

The graph shown in Figure 10 illustrates the alamarBlue response (percentage of reduced alamarBlue) for the PC12 cells following 24, 48 and 72 hour co-incubations with the QDs.

As seen in Figure 10, at 10^-7 ^M QD concentrations the toxicity is extremely high at all incubation times, and approached the levels of negative controls after only 48 hours. We can see the influence of the gelatine coating up to 24 hours as cell viability responses are significantly higher for the gel QDs compared to their non-gel counterparts. Notably, all responses are lower than the controls indicating that at this concentration the presence of any foreign entities generate a detrimental environment for the cells and result in high levels of cell death.

At 10^-8 ^M QD concentrations, we can now see a shift with respect to viability response. Initially after 24 hours, responses are comparable (note: orange non-gel QDs do show a slightly decreased response) between QD types and also to controls. This indicates that over this short incubation period, the cells are not significantly perturbed by the QDs at this concentration.

At 48 and 72 hours, the cell responses now mimic those seen for 10^-7^M concentrations and have dropped in comparison to controls; however, significant differences are noted between the two QD types. Responses for the gel QDs are considerably higher than those of the non-gel QDs and of note; the red QDs (whether gel or non-gel) are seemingly less toxic than the smaller orange QDs. This may be attributed to the fact that smaller QDs have been shown to penetrate further into cells than their larger counterparts. As nuclear pores are very small [45], nuclear staining of small "green" QDs and cytoplasmic localisation of larger "red" has demonstrated the size dependant nature of QD uptake [53]. Consequently, the smaller QDs may initiate deleterious cell reactions at far quicker rates than the larger ones.

Analysis of these responses at 48 and 72 hours reinforce the importance of the QD surface environment and the protective nature of the gelatine at this concentration. While the surface gelatine coating helps to reduce the toxicological impact of the QDs at 10^-8 ^M concentrations, at 10^-9 ^M we see the least amount of differences between QD types. Unlike previous concentrations, where alamarBlue responses decrease when comparing gel and non-gel QDs up to 72 hours, there is a certain amount of consistency when analysing the co-incubated QDs at 10^-9 ^M concentrations. There are no significant changes in cell response, across the total incubation period. We can also see that final 72 hour cell responses are actually comparable to those recorded for gel QDs at 10^-8 ^M. Throughout; all QDs types elicit responses below the levels of negative controls, however responses for gel QDs are far higher than non-gel QDs, indicating that even though their presence results in a certain level of toxicity, they are far less detrimental than their non-gel counterparts. As QDs are essentially a combination of toxic materials, their negative impact on cell health is to be expected, however as cell response seems to level off we can postulate as to the reasons for the induced QD toxicity.

The PC12s themselves can react to the presence of a foreign object, which may be the reason that overall QD cell responses are lower than the controls even after only 24 hours at low (10^-9 ^M) concentrations. From our data it is also notable that at 10^-9 ^M QD concentrations, the protective effect of gelatine coating was not obvious, with the sole exception of orange QDs at 24 hours. Thus, it can be argued that increases in cell viability at lower QD concentrations make it difficult for the protective effect of gelatine to be seen. CdTe QDs exert cytotoxicity characterised by decreases in the metabolic activity. The most common pathways involved in the toxicity of QDs are related to Reactive Oxygen Species (ROS). These free radicals act by activating different apoptotic pathways such as caspase-9-, caspase-3 and JNK [54]. Some studies have shown involvement of MAPK pathways *via *over-expression of TNF-α CxCl8 [55] or AP-1 and PTK pathways mediated by MMP2 and 9 over-expression [56]. Although there are different pathways involved, there is no obvious predilection for particular pathways in a particular cell line. A recent study with PC-12 cells has also shown involvement of reactive oxygen species (ROS) [45], where the authors have shown interactions of QDs with sub-cellular components and the detrimental effect of uncapped versus capped QDs [40]. This may indicate that the concentration of the leached atoms or reactive oxygen species even from non-gel QDs is so low at 10^-9 ^M as to minimally impact the cells beyond the toxicity induced by their very presence.

Throughout the assay, we can see a progressive increase in cell viability for gel compared to non-gel QDs, indicating that the gelatine must act as an effective barrier towards these processes occurring. While it does not prevent the resulting negative impact on the cells, the gelatine seems to effectively slow down the adverse effects of the QDs on cell viability, allowing for longer cell survival, thus enhancing imaging and analysis over elongated co-incubation times.

These results have been focussed on cell respiratory responses. Our next objective was to find out if the impact of the QDs remains the same for other cellular activities.

#### 2.2 PicoGreen Assay

PicoGreen kit Quant-iT™ dsDNA High-Sensitivity Assay Kit (Invitrogen) was used to quantify the amount of double stranded (ds) DNA in ng/μl.

**Figure 11:**
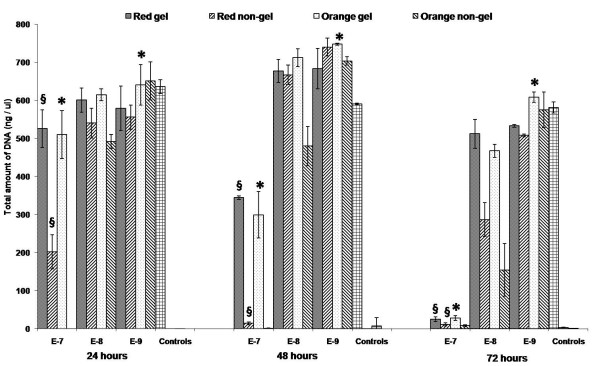
**PicoGreen histograms**. PicoGreen assay at 24, 48 and 72 hours illustrating the amount of DNA (ng/μl) measured from PC12 neurons following co-incubation with varying concentrations 10^-7-(-9) ^M of the gel and non-gel QDs. From left to right, controls [positive, negative, background] are also shown. §denotes examples of statistical significance due to effect of gelatine, * denotes examples of statistical significance due to effect of concentration using a one- way ANOVA (p < 0.05) by Tukey's mean comparison.

The graph shown in Figure 11 illustrates the total amount of DNA present (ng/μl) in live PC12 cells after 24, 48 and 72 hours of co-incubation with both the gel and non-gel QDs. This assay allows us to directly relate the impact of the QDs on the overall cell population.

At 10^-7 ^M QD concentrations, the histograms for the two QD types trend somewhat similarly to those seen for alamarBlue. Once again, responses never reach that of the control samples indicating the negative effect that the QDs have on this system. However, higher responses are once again recorded for the gel QDs after 24 hours and unlike the alamarBlue assay, the gel QDs show significantly higher results after 48 hours compared to the non-gel QDs. As before after 72 hours, both QD types elicit response similar to negative controls.

These data indicate that this assay seems to be more robust than the alamarBlue. This is an extremely sensitive assay to DNA concentrations and unlike the responses seen previously; there is an apparent shift in cell survival to longer co-incubation times. For example, responses for gel and non-gel QDs were comparable after only 48 hours with alamarBlue, while for PicoGreen this now occurs at 72 hours and this apparent shift continues as the concentrations are reduced.

As the QD concentrations are reduced to 10^-8 ^M, we can see that after 24 hours DNA responses are approaching comparability with positive controls. Small differences once again favouring the gel QDs can be seen and these continue up to 48 hours. Notably, as recorded before, the orange non-gel QDs begin to show the lowest response indicating their increased impact on cell survival.

Only at 72 hours do we see responses drop below positive controls and significant differences can be seen between the two QD types with once again the gel QDs producing higher responses. Thus, comparing the two assays at this 10^-8 ^M QD concentration, the shift to longer co-incubation times is clear indicating of increased cell survival rates and their ability to replicate for longer even in the presence of these toxic entities.

Similarly to the alamarBlue, there is a sense of consistency throughout the PicoGreen assay over all time points at 10^-9 ^M QD concentrations. DNA responses are comparable to positive controls and do not drop significantly even after 72 hours of co-incubation. This highlights the robustness of this cellular process to toxic influences at this concentration and also emphasizes the hormetic effect [2,57].

These results further corroborate those from the alamarBlue assay verifying that the nature of the QD surface (gel or non-gel) greatly influences their behaviour and the resulting viability of the cells.

The QD surface must be protected from the harsh intracellular environment if the cells are going to survive long enough to enable useful information about their behaviour and response to be gathered. The presence of gelatine on the QD surface clearly helps to reduce the impact of low intra-cellular pH ranges and the interactions of the various proteins present from breaking down the surface structure and releasing the "naked" toxic core atoms. Overall however the gelatine helps to nullify the toxic effects induced by the QDs; however the localisation of the QDs and their final destination must also play a role as there are variations in the impact that the different QD sizes and types have on each distinct cell response. This is quite significant and will require further investigation to fully determine and understand how changes in QD type, structure, surface functionality and concentration may impinge on the various cellular processes that occur during co-incubation.

#### 2.3 Proliferation ELISA BrdU

A Colorimetric Immunoassay was measured for the quantification of cell proliferation. This was based on the measurement of BrdU incorporation during DNA synthesis for the PC12 cells treated with different concentrations of gel and non-gel QDs. This cell proliferation allows us to extrapolate the healthy nature of the cells following co-incubation times of up to 72 hours. This assay is somewhat different from those previously examined as those cellular processes may still occur in cells that are not proliferating.

**Figure 12:**
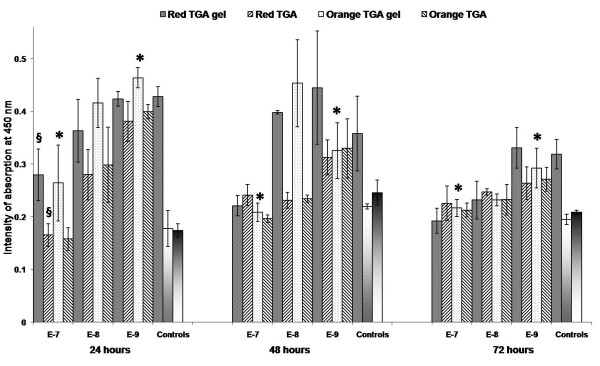
**ELISA BrdU histograms**. ELISA BrdU assay at 24, 48 and 72 hours illustrating (by intensity of absorption at 450 nm) the amount of cell proliferation following co-incubation with varying concentrations 10^-7-(-9) ^M of the gel and non-gel QDs. From left to right, controls [positive, negative, background] are also shown. §denotes examples of statistical significance due to effect of gelatine, * denotes examples of statistical significance due to effect of concentration using a one- way ANOVA (p < 0.05) by Tukey's mean comparison.

Figure 12 illustrates the measured response for cell proliferation upon co-incubation with the QDs after 24, 48 and 72 hours. Notably, negative and background control responses are significantly higher than those seen for alamarBlue and PicoGreen.

Initially after 24 hours at 10^-7 ^M QD concentrations, we can see a distinction between the less toxic gel and non-gel QDs however this levels off approaching negative controls at 48 and 72 hours. As the concentration drops to 10^-8 ^M, we can once again see the significant influence of the gelatine capping. At 24 and 48 hours the non-gel QDs are substantially more toxic approaching negative controls, while gel QDs maintain parity with positive controls. Little distinction is recorded at 72 hours illustrating the negative impact that prolonged co-incubation with the QDs has on cell proliferation at this concentration.

Similarly to previous assays, little distinction can be made between QD types as the concentration is reduced to 10^-9 ^M. After 24 hours, all QDs elicit responses in line with positive controls while after 48 and 72 hours, the red gel QDs once again showed the least detrimental effect on cell responses. Overall we can see a general trend towards a drop in cell proliferation with incubation time and the drop in responses for positive controls highlights the delicate nature of maintaining cell proliferation over extended co-incubation times. This also illustrates the extremely sensitive nature of this assay to external perturbation. Even though cell activity decreased during this assay application the results do show a similarity to those previously determined, albeit on a reduced scale.

## Conclusion

In conclusion, we have co-incubated and analysed PC12 cells over extended incubation times (up to 72 hours) with both gelatinised (gel) and non-gelatinised (non-gel) thioglycolic acid capped CdTe QDs. We have visually inspected QD localisation, cell morphology and behaviour at a range of QD concentrations (10^-7 ^- 10^-9 ^M). The presence of the QDs at 10^-7 ^M resulted in the death of all cells while at concentrations of 10^-9 ^M, the QDs were up taken primarily in the cytoplasm of the PC12s and did not initiate any detrimental effects.

The presence of gelatine on the QD surface was investigated by thermogravimetric analysis (TGA) which shows an additional 10% weight loss for the gel compared to non-gel QDs. Experiments conducted on the possible degradation of the QDs in the cell culture medium with serum have shown that quenching of the QD emission properties does occur due to protein-QD surface interactions. This does not induce a breakdown of the QD cores however, and indicates that any possible leeching of toxic core atoms must be induced by the internalisation of the QDs into the PC12 cells. We have also conducted experiments to enable us to empirically relate measured QD concentration to the actual weighed quantity of QDs present in mg/ml.

Utilising alamarBlue (cell viability) and Picogreen (DNA quantification) and ELISA BrdU (quantification of cell proliferation) assays we have measured and analysed cell response to co-incubations up to 72 hours with both gel and non-gel QDs. We have noted that throughout all our experiments, cell response varied in proportion to QD size, composition and concentration.

QD size significantly impacted measured responses. For the alamarBlue and PicoGreen assays at 10^-7 ^&^-8 ^M QD concentrations, the orange non-gel QDs consistently produced lower cell responses. This indicates that the increased cellular penetration of these smaller QDs resulted in enhanced adverse effects compared to their larger red counterparts. Notably, these effects were significantly nullified by the gelatine coating with similarly sized gel QDs producing higher response throughout.

Increased QD concentrations also lead to a decrease in all measured cell responses. Notably however, it is evident at all time points that the gelatine coating has a protective effect as cell viability and survival rates are significantly higher for gel compared to non-gel QDs. Elongation of co-incubation times (up to 72 hours) also highlighted the importance and the significance of the gelatine for QD surface protection. The assays have shown that the gel QDs were consistently less toxic than their non-coated counterparts at concentrations up to 10^-9 ^M. The presence of gelatine enables enhanced cell survival and proliferation at 10^-8 ^M compared to non-gel QDs, while its influence is negated at 10^-9 ^M concentrations over the longer co-incubation times. Thus, the 10^-8 ^M QD concentration appears to act as a threshold for the initiation of deleterious effects. At 10^-9 ^M concentrations, there appears to be a transition between the influences of QD surface structure (gel or non-gel) and QD concentration. The protective nature of the gelatine is countered by the drop in QD concentration and little variance was noted between the two QD types indicating that at this concentration the cells were unperturbed by the presence of either QD type.

## Materials and methods

### Chemicals and Reagents

PC12 cells (cancer cell line derived from a pheochromocytoma of the rat adrenal medulla) were used for this study. Dulbecco's Modification of Eagle Medium (DMEM) (Sigma-Aldrich) supplemented with 10% heat inactivated horse serum, 5% fetal bovine serum, 1% penicillin-streptomycin and, Trypsin-EDTA solution and all chemicals for QD synthesis were purchased from Sigma-Aldrich. Al_2_Te_3 _was purchased from Cerac Inc. AlamarBlue was purchased from Biosource International. A BrdU cell proliferation kit was purchased from Roche Diagnostics. Quant-iT PicoGreen DNA assay kit was obtained from Invitrogen. Permonax four-well chamber slide (Lab-Tek, Nalge Nunc International), Rhodamine-Phalloidin [Molecular Probes (Invitrogen)], DAPI (Vector Laboratories), Nunc tissue culture- treated 48-well plates were purchased from Biosciences and 96-well tissue culture plates were purchased from Sarstedt.

### Quantum Dot Synthesis

Note: all values denoted are initial concentrations and follows previously published procedures [49,58]. Millipore water (150 ml) was degassed by bubbling argon for approximately 1 hour. Cd(ClO_4_)_2_6H_2_O (3.22 g, [7.68 mmol]), TGA (thioglycolic acid) stabiliser (1.24 g, [13.46 mmol], 1.75 molar equivalents) was added and the pH was adjusted to 11.2-11.3 by the addition of a 2 M NaOH solution. For samples containing gelatine, 0.3 g was dissolved in 10 ml water by heating gently and added to the reaction mixture. H_2_Te gas, generated from Al_2_Te_3 _(0.56 g, [0.128 mmol]) *via *drop-wise addition of a 0.5 M H_2_SO_4 _solution was bubbled through the cadmium/thiol solution under a slow argon flow for approximately 10 minutes. Note: 100% reaction and carryover is assumed, and cadmium is always in excess for this experiment. The resultant, non-luminescent solution was then heated to reflux. Following the reflux process, fractions were precipitated *via *the addition of isopropanol and were stored at 4°C. The concentration of stock solutions used was approximately 2 × 10^-4 ^M [17] and were diluted by dissolving in de-ionised sterile water.

A Shimadzu UV-1601 UV - Visible Spectrophotometer was used to measure QD absorption while a Varian - Cary Eclipse Fluorescence Spectrophotometer was used to determine the fluorescence emission/photoluminescence (PL) spectra of QDs. A JEOL 3011 High Resolution Transmission Electron Microscope (HTREM) was used to image the QDs.

### Relating QD mass to concentration

Different batches of both gel and non-gel QDs were individually dried under rotary evaporation. The resulting powder was scraped from the flask and weighed before being re-dissolved in exactly 1 ml of purified water. The concentration was then determined [17], thus giving a relationship between QD molar concentration and the mass of QDs (mg/ml).

### Investigation of QDs in medium

Gel and non-gel QDs were diluted in cell culture medium to a final concentration of 10^-6 ^M. UV-Vis absorption and PL emission spectra were recorded at various time points up to 72 hours after addition.

### Thermogravimetric Analysis (TGA)

Samples of QDs (gel and non-gel) were dried on a rotary evaporator. The resulting powder was analysed by thermal gravimetric analysis on a Perkin Elmer Pyrus 1 instrument: it was heated from 30 to 900°C at a rate of 10°C/min and its weight was recorded continuously. The gelatine powder was also analysed.

### Cell Culture

PC12 cells, were cultured in medium (DMEM supplemented with 10% heat inactivated horse serum, 5% fetal bovine serum, 1% penicillin-streptomycin) @ 37°C and a 5% CO_2 _atmosphere. All the tissue culture plates and chamber slides were treated with 0.001% Poly-L-Lysine (PLL) for 24 hours.

### Cell Staining

Cells were seeded into four-well chambers at density of 10^5 ^cells/cm^2^. After 24 hours QDs were added (10% of amount of Medium) to make final concentrations in the range of 10^(-7)-(-9) ^M and the cells were incubated for different time periods from 24 - 72 hours. Cells were grown on 4 well Permonax Chamber slides in the presence of QDs. After the desired length of exposure, medium was removed and the coverslips were washed with 1% phosphate-buffered saline (BSA/PBS). Cells were fixed with 4% paraformaldehyde for 15 minutes and then washed 3 times with PBS. Then cells were permeabilized with permeabilizing solution (5 min, 0°C). Actin filaments of cytoplasm were labelled with Rhodamine Phalloidin (Molecular Probes (Invitrogen), at a 1:200 dilution with PBS for 15 minutes and again washed 3 times with PBS. Nuclei were labelled with Vectashield mounting medium with DAPI to preserve fluorescence and counter stained DNA with DAPI 1 μg/ml.

### Confocal Microscopy

An LSM 510 (Carl Zeiss, Jena, Germany) Confocal Laser Scanning microscope was used to examine QDs inside PC12 cells and its morphology.

Cell Imaging was carried out using a LSM 510 Inverted Confocal Microscope which is equipped with the following excitation lasers: (a) Argon Laser Excitation -wavelengths (λ_Ex_) = 458 nm, 488 nm, 514 nm, (b) HeNe1 - λ_Ex _= 543 nm, (c) HeNe1 - λ_Ex _= 633 nm and (d) Titanium Sapphire Tuneable Two-photon Laser tuneable from 710 nm to 1000 nm with a resulting excitation range of 355 nm to 500 nm.

Confocal laser scanning was carried out at laser scan speed of 7 with the Photomultiplier Tube settings adjusted to eliminate noise and saturation with the aid of the range indicator setting in the LSM 510 software. For image optimisation scan averaging was carried out on 8 scans per image.

Sequential acquisition was used to acquire the two colour images of the QDs in cells. For visualisation of the QDs, the samples were excited with the Argon 514 nm Laser and the microscope configuration was set up to capture the emitted fluorescence at 550 nm or 600 nm as desired. Differential Interference Contrast (DIC) or Nomarski Microscopy was used to visualise the cell morphology, and was carried out by using the HeNe1 488 nm laser with the Transmission Channel Detector selected and the DIC polariser and Nomarski prisms engaged. The two images were then over laid using the LSM 510 software.

Sequential acquisition was also used to acquire three colour images. Rhodamine phalloidin was excited using the HeNe1 543 nm laser and the emitted fluorescence was acquired at 575 nm. DAPI stain was excited with laser light at 390 nm (from the two photon laser tuned to 780 nm) and emitted fluorescence was acquired at 458 nm. The three separate images were over laid using the LSM510 software to make up the three colour images.

### AlamarBlue Assay

During cellular respiration, mitochondria take in oxygen and release CO_2_. During this process alamarBlue is substituted for molecular oxygen in the electron transfer chain and consequently becomes reduced. This reduction results in a change in both the colour and also the absorbance of the dye. These changes can be measured and are directly quantifiable against the number of healthy respiring cells present.

PC12 cells were seeded in 48-well micro-plates (Nunc) as triplicates. After 24 h, QDs were added (10% of amount of Medium) to make final concentrations in the range of 10^(-7)-(-9) ^M. Three different types of controls, namely: positive, negative and background were used throughout the study. Positive controls had cells with culture medium but without treatment with QDs. Negative controls were treated with QDs with culture medium and no cells. Background controls were cells treated with QDs but without culture medium. After 24 hours of treatment with QDs, the medium was removed and the wells were washed with HBSS. AlamarBlue solution was prepared by adding alamarBlue (Biosciences UK) and HBSS in the ratio of 1:10. 200 μl of alamarBlue solution was added to each well and the plates were incubated for 1 hour. 100 μl of reduced alamarBlue solution from each well was dispensed in a clear tissue culture 96 well plate. The Plate was analysed using a Wallac Victor Fluorescent Plate Reader. Absorbance was measured at lower wavelength of 550 nm and higher wavelength of 595 nm with a measurement time of 5.0 s. This was repeated with incubation periods of 48 hours and 72 hours.

### PicoGreen Assay

PicoGreen is a fluorescent stain that is highly selective for solubilised double-stranded DNA and is an extremely sensitive technique capable of nanogram DNA quantification. Unlike the non-destructive alamarBlue assay, a PicoGreen assay involves the freeze-thaw lysing of cells to analyse the quantity of dsDNA present. As the cells are washed to remove any dead cells before analysis, the assay only measures the DNA response from live healthy cells, thus allowing us to directly relate how the QDs impact cell survival rates.

The Quant-iT PicoGreen double-stranded DNA assay kit (Invitrogen) was used to assess DNA concentration. PC12 cells were grown in 48-well microplates (Nunc) as triplicates. After 24 h, QDs were added (10% of amount of Medium) to make final concentrations in the range of 10^(-7)-(-9) ^M. Three different types of controls, namely: positive, negative and background were used throughout the study. Positive controls had cells with culture medium but without treatment with QDs. Negative controls were treated with QDs with culture medium and no cells. Background controls were cells treated with QDs but without culture medium. After 24 hours of co-incubation with the QDs, the medium was removed and the wells were washed with HBSS. 200 μl of deionised double-distilled water was then added and the cells were lysed by freezing for 15 minutes at -80°C and thawing for 15 minutes at room temperature repeated 3 times. According to the assay kit a standard curve was then constructed. Final concentrations of the standards were 1000, 500, 100, 50, 25, 10, 5, and 0 ng/μl. 100 μl of lysed DNA solution of cells from each well were dispensed in a clear tissue culture 96-well plate. 100 μl of diluted PicoGreen solution were added to each of the test wells of 96-well plate. The Plate was analysed using a Wallac Victor Fluorescent Plate Reader by Fluorescence 485 nm/535 nm, 1.0 s protocol. Levels of DNA in each sample were calculated using the standard curve. This was repeated with incubation periods of 48 hours and 72 hours.

### Cell Proliferation ELISA BrdU

An ELISA BrdU (BrdU) assay involves the detection of 5-bromo-2-deoxyuridine, an analogue of thymidine, which is incorporated into the DNA of proliferating cells. Incorporated BrdU is labelled with a peroxidase-conjugated anti-BrdU antibody (anti-BrdU-POD). The amount of bound anti-BrdU-POD is quantified calorimetrically through exposure to a peroxidase substrate (3,3,5,5-tetramethylbenzidine [TMB]). TMB is acted upon by peroxidase to form a blue product. Upon addition of a stop solution (H_2_SO_4_), a yellow product is formed, which absorbs at 450 nm. The level of absorbance is directly related to the amount of cell division that has occurred during the course of the incubation period.

Cellular proliferation was measured using an enzyme-linked immunosorbent assay (ELISA) (supplied as a kit [Roche]). Cell Proliferation ELISA BrdU (Colorimetric) was performed according to the protocol in the manual of the kit. PC12 cells were grown in 96-well microplates (Nunc) as triplicates. After 24 h, QDs were added (10% of amount of Medium) to make final concentrations in the range of 10^(-7)-(-9) ^M.

Three different types of controls, namely: positive, negative and background were used throughout the study. Positive controls had cells with culture medium but without treatment with QDs. Negative controls were treated with QDs with culture medium and no cells. Background controls were cells treated with QDs but without culture medium. BrdU labelling solution was added to each well after 24 hours of adding QDs and incubated at @ 37°C and 5% CO_2 _atmosphere. The culture medium was removed and the cells denatured, and the anti-BrdU-POD added. This binds to the BrdU incorporated into cellular DNA. The level of incorporation is detected by means of a colorimetric substrate reaction. Quantification of the bound anti-BrdU-POD was accomplished by adding 100 μl TMB to each well and a further 20 minute incubation time at room temperature. 25 μl 0.1 M H_2_SO_4 _was then added, incubated for 1 minute and shaken at 300 rpm to stop the reaction. The Plate was analysed using the Wallac Victor Fluorescent Plate Reader (450-550 nm) protocol and measured absorbance for 2 minutes at room temperature. This was repeated with incubation periods of 48 hours and 72 hours.

### Statistical Analysis

Results of alamarBlue and PicoGreen assays were analysed using one-way analysis of variance (ANOVA). A ρ value of less than 0.05 for the ANOVA was considered significant. Error was expressed as a standard deviation.

## Abbreviations

QDs: Quantum Dots; CdTe: Cadmium Telluride; PC12: pheochromocytoma 12; NGF: nerve growth factors; TGA: Thioglycolic Acid; gel-QDs: gelatinised QDs; DNA: Deoxyribonucleic Acid; DMWM: Dulbecco's Modification of Eagle Medium EDTA; DAPI: 4: 6-diamidino-2-phenylindole; UV: ultraviolet; PL: photoluminescence; PLL: Poly-L-Lysine; BSA/PBS: Bovine serum albumin/phosphate-buffered saline; DIC: Differential Interference Contrast; HBSS: Hank's Balanced Salt Solution; TMB: 3,3,5,5-tetramethylbenzidine; HRTEM: High Resolution Transmission Electron Microscopy; FRET: Förster Resonance Energy Transfer; TGA: Thermogravimetric Analysis.

## Competing interests

The authors declare that they have no competing interests.

## Authors' contributions

BRP performed all cellular experiments and wrote the manuscript with SJB. SJB and VAG conducted the QD experiments. DC contributed with confocal imaging. YR, YG, NN, TJS designed the overall project and helped with data and manuscript revision. All authors read and approved the final manuscript.
